# Time-dependent behavior of the *Staphylococcus aureus *biofilm following exposure to cold atmospheric pressure plasma 

**DOI:** 10.22038/ijbms.2021.52541.11866

**Published:** 2021-06

**Authors:** Foad Fahmide, Parastoo Ehsani, Seyed Mohammad Atyabi

**Affiliations:** 1 Department of Molecular Biology, Pasteur Institute of Iran, Tehran, Iran; 2 Department of Nanobiotechnology, Pasteur Institute of Iran, Tehran, Iran

**Keywords:** Anti-infective agents, Biofilm, Extracellular polymeric, substances, Non-thermal atmospheric, pressure, plasma, Staphylococcus aureus

## Abstract

**Objective(s):**

Formation of *Staphylococcus aureus* biofilm leads to persistent infection in tissue or on exter-nal and indwelling devices in patients. Cold atmospheric plasma (CAP) is used for eradication of bacterial biofilms and it has diverse applications in the healthcare system. However, there is not sufficient information on the behavior of biofilms during the CAP exposure period.

**Materials and Methods:**

Pre-established *S. aureus* biofilms were exposed to CAP for 0 to 360 sec, then subjected to washing steps and sonication. Subsequently, biomass, number of colonies, vitality of bacteria, structure of colonies, size of produced particles, and viability of bacteria were evaluated by different assays including crystal violet, colony-forming unit, MTT, scanning electron mi-croscopy, confocal laser scanning microscopy, and dynamic light scattering assays.

**Results:**

The results showed that the strength of biomass increased in the first 60 sec, then decreased to less than no-CAP treated controls. Moreover, short CAP exposure (≤60 sec) ehances the fusion of the biofilm extracellular matrix and other components, which results in preservation of bacteria during ultra-sonication and washing steps compared with control biofilms. The *S. aureus* biofilm structure only breaks down following more CAP exposure (> 90 sec) and demolition. Interestingly, the 60 sec CAP exposure could cause the fusion of biofilm compo-nents, and large particles are detectable.

**Conclusion:**

According to this study, an inadequate CAP exposure period prevents absolute eradication of biofilm and enhances the preservation of bacteria in stronger biofilm compartments.

## Introduction

The majority of bacteria in nature and pathogenic milieu exist in biofilm forms. Biofilms are enclosed-matrix communities of microorganisms attached to a surface and gene expression is different from their planktonic counterparts, which offers protection in contrast to hostile factors. It could develop a tolerant phenotype by preventing the access of antimicrobial agents to the bacterial community (1). It has also been shown that bacteria in biofilm form are up to 1,000 times more resistant to antibiotics than their planktonic form (2). The Centers for Disease Control says about 1.7 million nosocomial infections are caused by bacterial biofilms; it has resulted in financial losses of $11 billion in the United States (3). Biofilms are capable of formation on medical implants, catheters, indwelling, and external devices (4), and cause bacteremia, osteomyelitis, skin infections, pneumonia, meningitis, and endocarditis (5). *Staphylococcus aureus *is an important pathogen that can develop a multilayered biofilm (6). The extracellular matrix (ECM) of *S. aureus *biofilms consists of e-DNA, proteins, and polysaccharide intercellular adhesion (PIA) which is made of poly-β (1-6)-N-acetyl glucosamine (PNAG) and is the main element of the exopolysaccharide matrix that encompass bacteria inside the biofilm and could cause biofilm-associated diseases (7). The related diseases are chronic osteomyelitis, chronic rhino-sinusitis, endocarditis, and orthopedic implant infection (8). Several influential antibiotics such as oxacillin, cefotaxime, and vancomycin have failed to penetrate *S. aureus *biofilm. Consequently, effective biofilm eradication is extremely difficult and is mostly cared for by surgery (5, 9). Due to direct connection of biofilm with nosocomial infections (10), there is a high demand for novel and effective biofilm-eradication technologies (11).

Plasma, the fourth state of matter, has been found in nature (12). The human-invented Cold atmospheric plasma (CAP) or Non-thermal plasma is a novel method, which acts as rupturing and lethal agent on a controlled region. The non-thermal feature of CAP (13) has been studied extensively against a broad spectrum of microbes in biofilm and planktonic forms (14). CAP generates photons, electrons, positively and negatively charged ions, atoms, free radicals, and excited or non-excited molecules under a constant supply of energy in a non-thermal condition (13). Therefore, exposure of CAP, generated by different gases such as Ar, He, O2, N2, on biological surfaces induce physical and chemical changes to the biomolecules such as DNAs, proteins, and polysaccharides (15). 

So far, many studies have been conducted on the effects of CAP on a wide range of pathogens (14, 16, 17) and had also exhibited anti-biofilm activity (16, 18, 19). Moreover, the destruction of biofilm occurs through two mechanisms; 1) intense cell membrane damage and following bacterial eradication in biofilms, 2) affecting biofilm structure and disconnecting the biofilm from a solid surface by damaging the ECM (20).

CAP has the potential in the therapeutic on humans and heat-sensitive materials In comparison with the traditional methods (high pressures and temperatures, irradiation, and chemical agents) (21-24). Moreover, it is available in a completely everyday and handheld way (25), and the apparatus could be made by professional supervision at the laboratory (26,27). Because of its restricted submissions, it does not harm the total microbiota (12). Therefore, determination of parameters for effective plasma ramifications on bacteria especially in the form of biofilm is extremely vital.

In the following study, a cold plasma jet source with helium input gas was managed directly on *S. aureus *biofilms produced in a 96-well plate (28). The effects of CAP on bacteria in biofilm and its structure were determined using different approaches to provide a more comprehensive analysis.

## Materials and Methods

***Bacterial strain and culture***

*S. aureus *(ATCC 6538) strain was used to prepare the inoculate. A single bacterial colony from a blood agar plate was selected and grown in 10 ml Trypticase Soy Broth (TSB) without glucose (Sigma) and incubated under orbital agitation (120 rpm) at 37 °C for 24 hr. 

***Cold atmospheric plasma treatment of biofilms***

The device consisted of a power supply including electrodes, which worked with a 15 MHz frequency, 12 kV, and 12 W output power radio. CAP was generated through ionization of pure He with constant (1 l/min) discharge with 1.5 bars atmospheric pressure (27). 

***Microplate biofilm development and CAP treatment***

The overnight bacterial suspension was homogenized by a 1-minute vortex and diluted to ~10^5^ cells/ml in TSB media containing 1% glucose (TSBG) (29). Then 250 µl of bacterial culture was added in each well of a 96-well polystyrene flat-bottom cell culture plate ) Corning) in a biological safety cabinet and placed in a stationary incubator at 37 °C for 48 hr. The consumed TSBG was removed by a multichannel pipette, and the attached biofilms gently rinsed using adequate wash steps, which means thrice to avoid false-negative wells (29) by 300 µl pre-warmed sterile phosphate-buffered saline (PBS) to remove non-adherent and sedimentary bacteria, then subjected to CAP exposure. 

The CAP discharge was conducted to the bottom of the wells of microplates by 30-mm distance from the plasma jet nozzle in an unceasing exposure run. The standard condition including ambient temperature and a non-stop exposure run was performed. Moreover, an empty well was left between CAP-treated ones for preventing the effect of plasma on neighboring wells. The biofilms were surveyed in triplicate at 0, 30, 60, 90, 120, 240, 360 sec, in sterile conditions (16, 30, 18, 31). The non-treated wells were considered as control.

***Biomass quantification of CAP-treated biofilms by crystal violet assay***

Following CAP treatment 100 μl of fresh 0.1% CV (Merck) (w/v) solution was poured into each well for 15 min. Then, it was gently immersed in distilled water four times to remove the unbound dye. Following fixation with 150-μl methanol for 20 min, it was left to air-dry in an inverted position at room temperature. The stains were adsorbed by the biomass dissolved in 150 μl of 33% acetic acid without rotation at ambient temperature. Then, transferred to a new microplate and the absorbance at A430 nm (Epoch-2 Microplate reader - BioTek Instruments, USA) was determined. 

***Characterization of CAP-treated biofilms***

The CAP treated and untreated wells of the microtitre plates were washed three times and 100 μl PBS was added to each well. Then they were insulated with Parafilm (Bemis M PM992 Laboratory Wrapping Film) and subjected to ultra-sonication at 35 kHz for 10 min in a 10 °C water bath (Bandelin Sonorex Digitec DT 31). 100 µl of the suspended biofilm was added to 900 µl of TSB. The diluted suspension was used to determine the number of growing bacteria, size of the particles, and viability of bacteria by CFU, DLS, and MTT experiments, respectively as mentioned below. 

***Quantification of CFU of CAP treated biofilms***

100 µl of ultra-sonicated biofilm suspensions were serially diluted and the four last dilutions spread on Mueller-Hinton agar by cotton swap and incubated for 24 hr at 37 °C.

***Quantification of metabolic activity of bacteria in CAP treated biofilm by MTT assay***

One hundred μl of the ultra-sonicated biofilm suspension was added to each well. MTT reagent (yellow tetrazole, Bio Basic Inc, Canada) was poured into each 96-well plate to a final concentration of 0.5 mg.ml^-1^ and incubated at 37 °C. Following 4 hr of incubation, the wells were treated with 1:1 DMSO) for 15 min with agitation at room temperature to dissolve purple formazan crystals and absorbance was measured at OD 570/630 nm (32) by a Microplate reader (Epoch-2 -BioTek Instruments, USA) (33). The background (DMSO-MTT) was subtracted from all values. 

***Determination of the particle size distribution by DLS***

The Particle size distribution was determined by dynamic light scattering (DLS). The CAP-treated biofilms were resuspended in PBS by ultra-sonication and diluted before the DLS running (34) (ZetaSizer ZEN 3600, United Kingdom).

***SEM and CLSM imaging***

An amount of 2.5 ml TSBG containing equal 105 CFU.ml ^-1^ of *S. aureus *was poured into each well of flat bottom 24 cell culture plates (Corning™ Costar™) containing autoclaved coverslips (15 mm round cover glass, Cell treat Scientific) and incubated at 37 °C for 48 hr. For SEM, the glasses were gently rinsed three times with 3 ml PBS and placed angled in sterile chambers. Next, the glasses were air-dried and fixed with 2.5% glutaraldehyde (v/v) in PBS (pH 7.2), 4 °C for 16 hr. Then, the glasses were dehydrated by increasing ethanol concentrations (20%, 40%, 60%, 80%, and 96%) at ambient temperature, each for 10 min and next placed into a desiccator intended for gold coating procedure. The coverslips were mounted on aluminum SEM stubs. Finally, the images captured by scanning electron microscopy (NOVA NANOSEM 450 FEI, Stain Laboratory- Department of Physics at University of Tehran , Tehran, Iran)(35).

For CLSM, the biofilm containing glasses were stained with 100-μl propidium iodide (PI) and fluorescein isothiocyanate (FITC) (Sigma-Aldrich) both at 1 mg.ml^-1^ concentration and placed for 15 min at 37 °C in the dark. Then they were washed gently three times with 3 ml sterile water. The images were monitored using (CLSM 510) Zeiss confocal laser scanning microscope with excitation-emission wavelengths set at 488-530 nm, respectively. The captured images (25–50x magnification) were analyzed by Zeiss Efficient Navigation (ZEN) 2009 software (Carl Zeiss, Germany) to determine the effect of treatments on bacterial biofilms (Core Facility, Pasteur Institute of Iran) (36). 

***Statistical analysis***

All experiments were conducted three times in triplicate and each data bar is the mean ±SD of three independent experiments (Figures 2–5). Statistical analysis and graphs were carried out with Prism (Version 8.0. for Windows, Graph Pad, USA), and the one-way ANOVA test was performed. A P-value of ≤0.05 was considered significant.

## Results

The CV absorbance value in the CAP treated biofilms

Crystal violet stains bacterial cells but not the slimy material (37). The absorption value of the control biofilms was constant during 360 sec in the experiment; however, for the CAP treated wells, a sharp peak emerged in 60 sec compared with control. Following 120 sec of CAP discharge, the CV absorbance reaches around the control absorption value. The biofilm-deterioration effect of plasma discharge began at 90 sec and lasted up to 120 sec, and as a result, the amount of CV absorption decreased compared to the control. Following more exposure, the CV absorbance values remained constant. The detailed effect of CAP on a 48-hr biofilm was illustrated in (Figure 2).

***Results of CFU and MTT determination of CAP treated biofilms***

Following CAP treatment, ultra-sonication was applied to breakdown the weak interactions of biofilm either with the surface of the microplate or between the biofilm components. CFU was determined and a peak at 60 sec was demonstrated. Following more CAP exposure (>120 sec) CFU dropped significantly. Furthermore, at >240 sec and 360 sec, 3*log reduction was recorded. 

The metabolic activity of bacteria of the CAP treated biofilms compared with untreated (zero time) was determined by MTT assay (Figure 4). The result demonstrated a correlation between CFU and MTT assays (Figures 3 and 4). The absorbance at 570 nm of 0 sec for MTT assay was 0.157±0.006, then reached 0.257±0.026 in 30 sec and increased to 0.289±0.017 in 60 sec, which was the highest level. Next, it dropped to 0.201±0.037 in 90 sec and 0.042±0.048 at 120 sec. Later, at 240 sec and 360 sec, the absorbance value was equal to blank.

***DLS analysis of CAP treated biofilms***

Following CAP treatment, the particle size distribution of suspension was determined by Nano-sizer (Figure 5). The results showed that the size of the particles increased following 30 sec ( ~ 1.866×10^4^ nm) and 60 sec ( ~ 4.66×10^4^ nm) CAP exposure compared with the original average size ( ~ 0.203×10^4^ nm) in no CAP treated controls. Following more CAP exposure, the size of the particles decreased (~ 3.35×104 nm) at 90 sec and then at 120 sec (~2.07 ×10^4^ nm). Eventually, the average size reached 0.6316×10^4^ nm at 240 sec and ~ 0.3652 ×10^4^ nm at 360 sec of CAP exposure.

***SEM Analysis of the CAP treated biofilms on glass slides***

The SEM of the CAP treated biofilms showed the detachment of biofilms following 90 sec of exposure compared with the untreated controls at 1500X magnification (Figure 6). Then, aggregation of bacteria in 30 sec and more interactions such as fusion in 60 sec were demonstrated in magnification of 24000X. Following 90 sec of exposure, the perforation of bacteria changed the morphology of *S. aureus *(24000X).

***Visualization of CAP-treated biofilms by CLSM***

The live cell imaging of CLSM showed compared with the control sample the CAP exposure decreases the interactions of proteins with green FITC while interaction of red PI with exposed DNA increases and the latter confirms the abundance of dead bacteria. Departed large pieces of biofilm and an enormous amount of dead cells at 120 sec is evident. 

**Figure 1:**
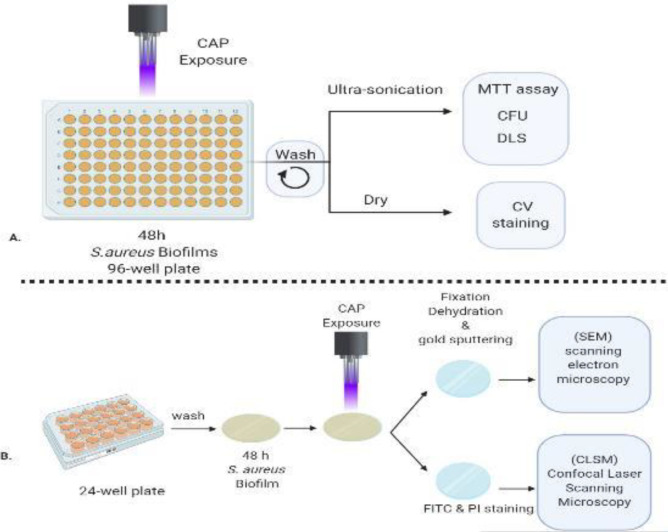
Graphical abstract of the experiments; A. biofilm formation on 96-well micro Plate. B. Biofilm formation on round glass coverslips

**Figure 2:**
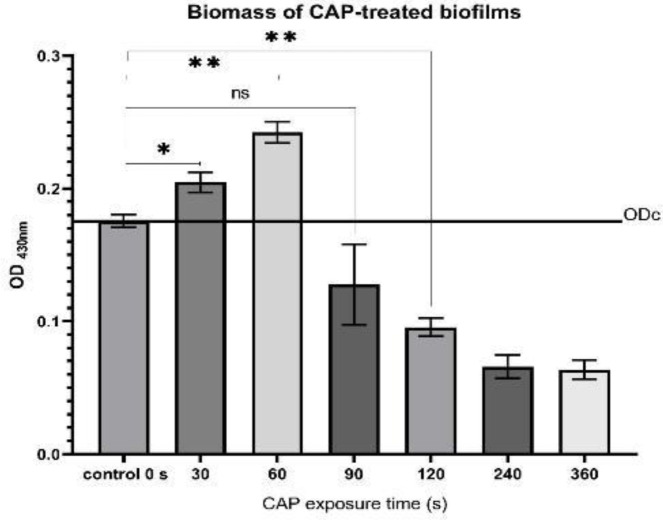
Biomass determination of biofilm exposed to CAP in varied time courses using CV assay: Control: no-CAP exposed biofilms (time zero)

CAP: Cold atmospheric plasma; ODc: OD of control for comparison, ns: not significant

**Figure 3:**
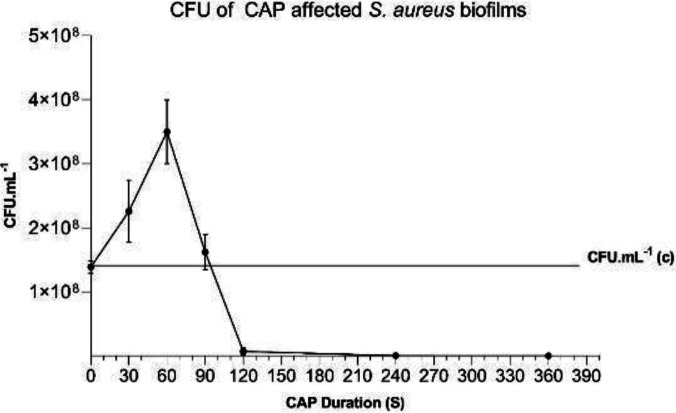
CFU of *Staphylococcus aureus* CAP affected biofilms (48-hr) compared with no-CAP treated at the time of zero as control

CFU:Colony forming unit; CAP: Cold atmospheric plasma

**Figure 4:**
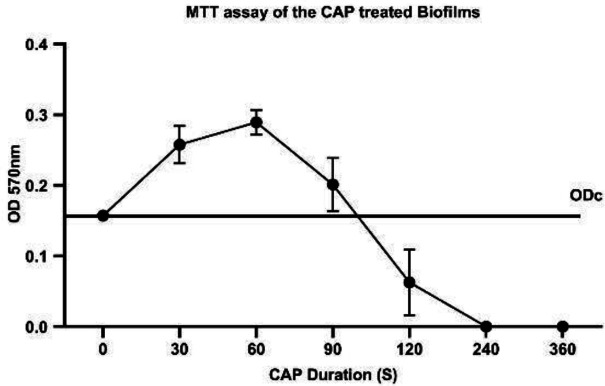
Bacterial viability of CAP treated biofilm by MTT assay among increasing exposure time of CAP compared to time zero as control

CAP: Cold atmospheric plasma; MTT: Metabolically active bacteria; ODc: OD of control for comparison

**Figure 5:**
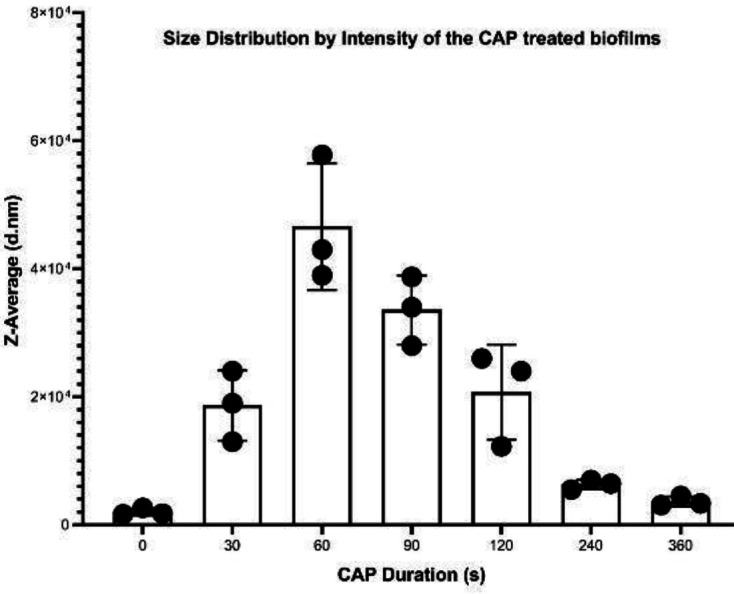
Size Distribution of particles based on Z average following different periods of CAP treatment: zero (control), the 30 sec, and 60 sec, 90 sec, 120 sec, 240 sec, and 360 sec of CAP exposure

CAP: Cold atmospheric plasma

**Figure 6:**
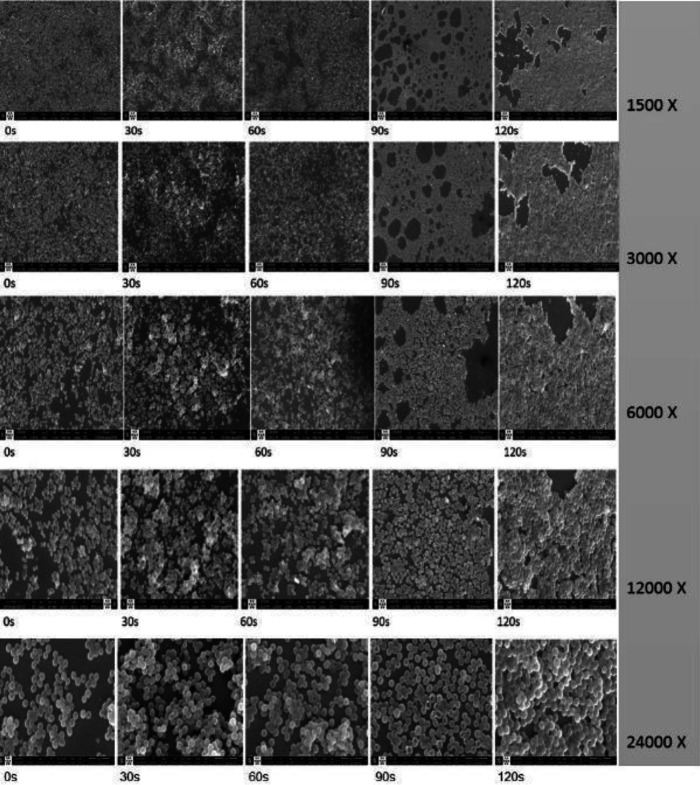
SEM Morphology imaging of the glass-developed *Staphylococcus aureus *biofilms resulting in CAP treatment at increasing time exposures of (0, 30, 60, 90, and 120 sec) in different magnification. Zero seconds is non-CAP ex-posed biofilm (control)

CAP: Cold atmospheric plasma

**Figure 7:**
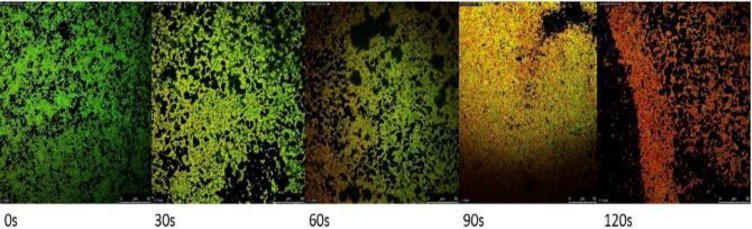
CLSM, Real-time structure analysis of *Staphylococcus aureus* glass-developed biofilms following FITC and PI staining. CAP treated biofilms captured at increasing time exposures of (30, 60, 90, and 120 seconds). Zero sec-onds is non-CAP exposed biofilm (control), The bars are 0-50 µm

CLSM: Confocal laser scanning microscopy; FITC: Fluorescein isothiocyanate; CAP: Cold atmospheric plasma

**Figure 8:**
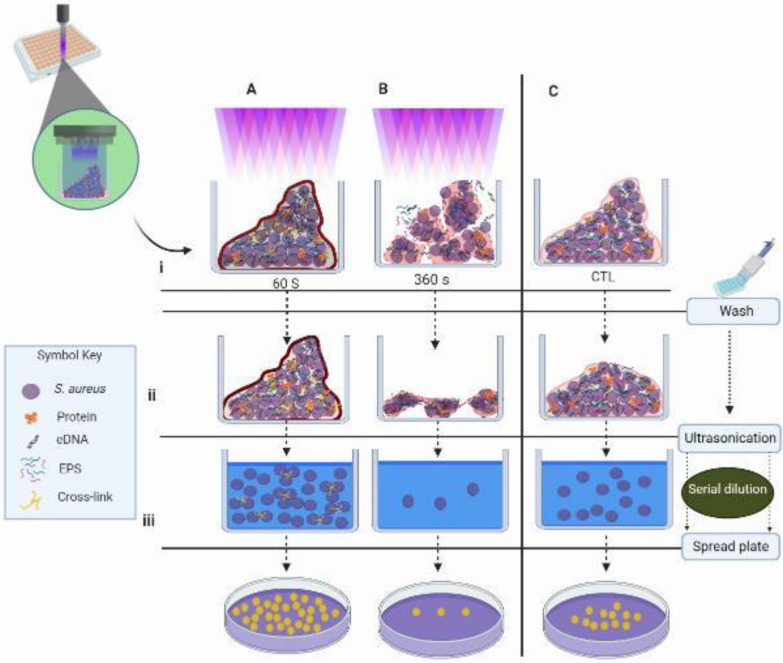
Schematic image of the effect of CAP exposure duration on *Staphylococcus aureus *biofilm inside the 96-well plate. i. Biofilms following CAP exposure (A) The 60 sec CAP, (B) the. 360 sec CAP.), and non-exposed (C) control. ii Wash step: the loose pieces removed. iii. The ultra-sonication, the remained biofilms suspended in the superna-tant. The serial dilutions of the supernatant of each treatment spread on the plate

CAP: Cold atmospheric plasma

## Discussion

CAP treatment has effects on biofilm components, on the biofilm as a whole compartment and individual bacterium. CV experiment was used to define CAP treatment effect on biomass as a compartment. The results showed that CV optical density increases in the first 90 sec with a peak of around 60 sec (Figure 2). This is due to two factors: 1. An increase in intra-biofilm interaction stabilized the biofilm during washing steps compared with untreated biofilm, and 2. More biofilm interactions with CV. The first effect has also been shown by Fahmey et al. (38) by confirming the increase in the size and stability of liposomes following treatment with CAP. However, the second effect is due to modification of biofilm components (as a surface catalyst) by the reactive elements produced by CAP treatment; thus, affecting the chemistry of catalysts of biofilm and resulting in more interaction with positive charges of CV molecules (39). However, with more exposure, one or both mechanisms interfered and destruction of the biofilm occurred (SEM and CLSM results, Figure 6 and 7), and consequently, biofilms were washed away. Accordingly, the CV adsorption decreased (Figures 2 and 8).

Each washing step declined the number of loos bacteria or biofilms (29) including the control wells. Therefore, when CAP exposure reinforces the integrity of biofilm and its resistance to the wash step, the amount of biofilm in negative control decreases. Thus, in CAP exposed wells of 60 sec the increase in the interactions between the biofilms augments the interacted CV (Figure 8).

 In the following experiments, the CAP-treated biofilms were subjected to ultra-sonication for enhancing the destruction of loose bonds to demonstrate the CAP-enforced interactions using CFU, MTT, and DLS assays on the particles in supernatants. The result of the 60 sec of CAP treatment showed a rise in CFU and MTT compared with controls. The increase in CFU was not due to bacterial duplication, which generally takes about 24 min (40) but is because of an increase in the stability of biofilm as mentioned before (Figures 3 and 8). The short CAP treatments (≤ 60 sec) induce extra intra/inter component interactions that made the biofilm tolerant to the wash step and kept biofilm components together and more growth of brought forward bacteria compared with the control. Fahmey *et al*. also saw this effect. They showed application of CAP on bilayer lipid membrane results in larger and more stable compartments (38).

Sonication in the present study affects the biofilm in two ways, separation of biofilm from polystyrene wells of microplates, which is mainly based on hydrophobic interactions (41), and breaking down the inter /intra biofilm interactions. This enhances the releasing of the bacteria including the dormant to the supernatant, promoting the bacteria to access leftover nutrients and oxygen during the next 4 hr which results in metabolically active individuals. 

Moreover, ultra-sonication was conducted (35 kHz) at 10 °C for 10 min. This is in accordance with Monsen et al. that have shown 88% of *S. aureus *survived during sonication at 40 kHz and low temperatures (42). Dudek et al. have also shown that a low temperature of 6 °C preserves the viability of *S. aureus *during the first 10 min of sonication. (43)

Considering the above statements, more bacteria are brought forward in enforced biofilm compartments induced by 30 sec and 60 sec of CAP exposure compared with the negative control. Consequently, more metabolic activity was measured by MTT assay (Figures 4 and 8). However, when more than 60 sec of CAP was exposed due to more destruction of biofilm and eradication of the bacteria, MTT and CFU decreased (Figures 3, 4, and 8). These results are similar to Alkawareek’s study which showed that following 120 sec of exposure to a gas mixture (0.5% oxygen and 99.5% helium), the *S. aureus *bacteria in biofilms are eradicated. The difference between the killing rates of Alkawareek’s study with this study could be due to the type of input gases; as the presence of O2 induces a stronger antibacterial effect due to produced Reactive oxygen species (ROS), such as atomic oxygen, ozone, peroxide, superoxide, and hydroxyl radicals. 

Moreover, in Alkawareek’s study, there is a plateau in the surviving curve of bacteria around 30 sec of exposure time (16). They did not discuss the reason; however, it may be caused by the factors presented above including the fusion of biofilm components that decreases the CAP eradication effect.

Researchers studied the influence of helium plasma jet on a 12 hr-formed *S. aureus *biofilm. They employed ~ 1×107 cell inoculum in TSBG (2 ml) and prepared the biofilms on borosilicate slices (6 mm diameter) then, by 10-min CAP exposure biofilms were eliminated by 3.06 in CFU log (more than 99.9% decrease)(19). Nevertheless, they did not explore the CAP effect in shorter times. Furthermore, we reached the three*log reduction at >240 sec CAP application on 48-hr biofilm.

The DLS experiment was applied to biofilm to determine the effect of CAP on the physical characteristics of particles. Considering the size of each *S. aureus *to be about 1-1.5 µm (44) DLS has shown that the mean size of particles in control wells, following ultra-sonication is about 2.030 µm, and at least two bacteria in each particle are involved (Figure 5). However, following 60 sec exposure the large particles of 46.600 µm, were distinguished. This also confirms the formation of larger particles, that due to more intermolecular bindings (fusion-like compartments) became resistant to ultra-sonication or pipetting compared with control. SEM confirmed the presence of such compartments at 60 sec using 24000X magnification (Figure 5). 

SEM was performed to reveal the actual condition of the CAP-treated biofilms (Figure 6). In 1500X magnification, the loss of biofilm integrity and the spread of the patches in the biofilm lawn following 90 sec of CAP exposure were seen. However, in 24000X magnification, the multilayer *S. aureus *biofilm was preserved in 30 sec and 60 sec of CAP treatment (Figure 6) compared with the control. The fusion of the biofilm components is shown in Traba and Liang’s study (45). They have applied CAP using argon gas on 24 hr and 7-day old biofilm for 5–10 and 1–60 min exposure. However, in the present study, the fusion of biofilm components was noticed following 60 sec of exposure on 48 hr biofilm. The difference could be due to the age of the biofilm, plasma device, exposure time, power and flow rate of gas, type of the gas applied, and missing the event because of the long interval (5 min) of CAP treatments in Traba and Liang study (20). Following more exposure, the fused multilayer structure is destroyed, much larger fragments were detached (1500X) from the glass-developed biofilm in the 90 sec and 120 sec as islands (Figure 6). Besides, the bacteria are losing their integrity due to the pores formed in the cell membrane as shown in 90 sec and 120 sec in 12000X and 24000X (Figure 6). 

CLSM was performed to detect the viability status of treated biofilms. As CAP exposure increases, the green color of FITC decreases which indicates fewer interactions due to emerging denatured proteins. However, the red color of the PI that interacts with the exposed DNA following cell membrane damage due to long CAP exposure increases as a sign of mortality of bacteria (Figure 7). 

In this study, the effect of CAP exposure on 2-day old *S. aureus *biofilm is shown. The biofilm goes through different stages of re/de organization of its structure following exposure to plasma. These modifications are based on the molecular interactions due to reactive compounds produced by CAP. In the first 60 sec of discharge, the molecules of biofilm components activated and induced inter/intra-molecular interactions. Thus, building up a multilayer biofilm structure that absorbs more CV molecules and retains its structure following wash steps compared with untreated biofilm. This process results in more CFU in the first 60 sec. Moreover, due to the destruction of biofilm by ultra-sonication, access of the brought forward and suspended dormant cells to oxygen and the leftover nutrients increased the number of metabolically active bacteria (MTT) compared with control biofilm (Figure 6). Further CAP exposure (>60 sec) demolishes the multilayer structure and kills bacteria as shown by SEM and CLSM, respectively. Accordingly, the results showed that at long exposure time (>120 sec), the bacteria destroy biofilms and eradication occurs. 

## Conclusion

CAP presents a non-toxic treatment of heat-sensitive surfaces, and in comparison with traditional methods (high pressures/temperatures, irradiation, and chemical agents) is less or even not harmful. We confirmed that helium plasma jet could be applied to damage the biofilm. However, short and long-term CAP exposures have different consequences. The short exposure time could fuse the biofilm components and keep the source of infection in its microenvironment and if biofilm structure is impaired the active bacteria could be released, However, the long-term exposure >240 sec destroys the biofilm and bacteria. Therefore, regarding the benefits of CAP for medical use, its application should be supervised with care.
